# Validation of the GenesWell BCT Score in Young Asian Women With HR+/HER2− Early Breast Cancer

**DOI:** 10.3389/fonc.2021.588728

**Published:** 2021-02-23

**Authors:** Mi Jeong Kwon, Jai Min Ryu, Soo Youn Cho, Seok Jin Nam, Seok Won Kim, Jeeyeon Lee, Soo Jung Lee, Ji-Young Park, Ho Yong Park, Sungjun Hong, Kyunga Kim, Jinil Han, Youngho Moon, Young Kee Shin, Jeong Eon Lee

**Affiliations:** ^1^Vessel-Organ Interaction Research Center, College of Pharmacy, Kyungpook National University, Daegu, South Korea; ^2^Research Institute of Pharmaceutical Sciences, Kyungpook National University, Daegu, South Korea; ^3^Department of Surgery, Samsung Medical Center, Sungkyunkwan University School of Medicine, Seoul, South Korea; ^4^Department of Pathology and Translational Genomics, Samsung Medical Center, Sungkyunkwan University School of Medicine, Seoul, South Korea; ^5^Department of Surgery, School of Medicine, Kyungpook National University, Kyungpook National University Chilgok Hospital, Daegu, South Korea; ^6^Department of Oncology/Hematology, School of Medicine, Kyungpook National University, Kyungpook National University Chilgok Hospital, Daegu, South Korea; ^7^Department of Pathology, School of Medicine, Kyungpook National University, Kyungpook National University Chilgok Hospital, Daegu, South Korea; ^8^Department of Digital Health, Samsung Advanced Institute for Health Sciences & Technology, Sungkyunkwan University, Seoul, South Korea; ^9^Statistics and Data Center, Research Institute for Future Medicine, Samsung Medical Center, Seoul, South Korea; ^10^R&D Center, Gencurix Inc., Seoul, South Korea; ^11^Laboratory of Molecular Pathology and Cancer Genomics, Research Institute of Pharmaceutical Sciences and College of Pharmacy, Seoul National University, Seoul, South Korea; ^12^Department of Molecular Medicine and Biopharmaceutical Sciences, Graduate School of Convergence Science and Technology, Seoul National University, Seoul, South Korea

**Keywords:** GenesWell BCT assay, BCT score, prognostic value, predictive value, young breast cancer patients, HR+/HER2− early breast cancer

## Abstract

**Background:**

The prognostic or predictive value of commonly used multigene assays in young patients with hormone receptor-positive (HR+), human epidermal growth factor receptor 2-negative (HER2−) early breast cancer is unclear. In this study, we assessed the prognostic value of the GenesWell BCT assay according to age group.

**Methods:**

We identified patients with pN0-1, HR+/HER2− breast cancer in a prospective cohort of women who underwent surgery between 2005 and 2017. The GenesWell BCT assay was performed on tissue samples from selected patients. Distant metastasis-free survival (DMFS) and disease-free survival (DFS) were compared between the risk groups assigned by the BCT score.

**Results:**

A total of 712 patients were eligible for analysis. The median follow-up time was 7.47 years. The BCT score was prognostic in patients aged ≤50 years (n = 404) and those aged >50 years (n = 308). In both age groups, the 10-year DMFS and DFS rates for patients classified as high risk by the BCT score were significantly lower than those for patients classified as low risk. A multivariate analysis revealed that the BCT score was an independent prognostic factor for DFS in patients aged ≤50 years (hazard ratio, 1.28; 95% CI, 1.05–1.56; *P* = 0.015), as well as those aged >50 years.

**Conclusion:**

The BCT score could be used to identify low-risk patients who will not benefit from adjuvant chemotherapy to treat HR+/HER2− early breast cancer regardless of age. A further prospective study to assess the prognostic and predictive value of the BCT score is required.

## Introduction

Young age at diagnosis is a negative prognostic factor for patients with early breast cancer, particularly those with hormone receptor-positive (HR+), human epidermal growth factor receptor 2-negative (HER2−) breast cancer (1–3). Accordingly, young age is often used as an indication for adjuvant chemotherapy, and some studies have reported that most (>80%) young patients with early breast cancer receive adjuvant chemotherapy (4, 5). However, HR+/HER2− breast cancer patients benefit less from chemotherapy than HER2+ or triple-negative breast cancer patients (6), and young age alone should not be a reason to expand chemotherapy indications in early breast cancer (7, 8) Therefore, it is important to identify patients who will not benefit from chemotherapy to avoid unnecessary chemotherapy in young patients with HR+/HER2− breast cancer.

Several multigene assays, such as MammaPrint (9) and Oncotype DX (10), have been developed to predict the risk of recurrence or response to adjuvant chemotherapy in early breast cancer. However, most of those assays were developed using data mainly from postmenopausal women in Western countries (11, 12), and recent prospective clinical trials (e.g., TAILORx for Oncotype DX and MINDACT for MammaPrint) also included only a small number of young breast cancer patients (13, 14). Few data are available regarding the prognostic or predictive value of the commonly used multigene assays in young breast cancer patients. Moreover, recent TAILORx results show that the Oncotype DX recurrence score (RS) has different treatment implications for patients aged ≤50 and those aged >50 years (14), and they further revealed that the clinical risk classification in combination with RS provided prognostic information for identifying young patients who could benefit from chemotherapy (15). Those findings raised concerns about the value of existing multigene assays for deciding whether to use adjuvant chemotherapy in young breast cancer patients.

Another concern is that the median and peak age of breast cancer patients in Asian populations, including South Korea, Japan, Singapore, and Taiwan, is younger than in Western countries (16, 17). Breast cancer in young Asian women has distinctive disease characteristics compared with that in Western countries (18, 19). Therefore, it is particularly important to elucidate the reliability of multigene assays in young Asian women with breast cancer.

The GenesWell Breast Cancer Test (BCT) is a prognostic assay that predicts the risk of recurrence in patients with HR+/HER2− early breast cancer (20). This assay was developed using data from Asian patients, including a higher percentage of young patients than was used for previous assays. A recent study comparing the BCT score and the Oncotype DX RS for risk classification found that the concordance between the two risk scores was low in women aged ≤50 years, suggesting the need to find more adequate tests in that population (21). In this study, we analyzed the distribution of BCT scores by age group and assessed its prognostic value in patients aged ≤50 years and >50 years.

## Materials and Methods

### Patients and Tumor Samples

A total of 3,289 patients with T1–3, N0–1, HR+/HER2− early breast cancer who underwent surgery at SMC between July 2005 and December 2017 or at KNUH between January 2009 and December 2017 were screened in a prospectively collected patient cohort, and their clinical information and survival data were collected. Clinical information included their age at operation, tumor size, pathologic nodal (pN) status, pathologic stage according to the 7th edition of the American Joint Committee on Cancer classification, histologic grade, nuclear grade, lymphovascular invasion (LVI), multiplicity, Ki-67 (%), estrogen receptor (ER)/progesterone receptor (PR)/HER2 status, and use of hormone therapy or chemotherapy. ER/PR/HER2 status was obtained from the pathological report. ER and PR results determined by immunohistochemistry (IHC) were considered positive when at least 1% of tumor cells showed nuclear staining, according to American Society of Clinical Oncology/College of American Pathologists guidelines (22). HER2 was considered positive if ≥10% of tumor cells showed 3+ staining by IHC or 2+ staining by IHC with amplification using fluorescent or silver *in situ* hybridization (23).

Patients lacking clinical information or survival data, and those with short follow-up duration (≤12 months) were excluded from the sample. We stratified the included patients by age group and nodal status (pN0 or pN1) and then selected patients from each age group (31–40, 41–50, 51–60, >60 years) so that the ratio of patients with pN0 and pN1 tumors in each age group was 2:1. All patients in their 20s or 70s were included. The quantity and quality of formalin-fixed, paraffin-embedded (FFPE) tumor samples from the selected patients were evaluated, and only patients with sufficient FFPE tumor samples were then used to test the GenesWell BCT assay. In case of multiplicity, paraffin block from the largest mass was used to assess the BCT score.

### GenesWell BCT Assay

Total RNA was isolated from the FFPE samples, and the GenesWell BCT assay was performed as previously described (20). The BCT score was calculated from the relative expression values of six prognostic genes (*UBE2C*, *TOP2A*, *RRM2*, *FOXM1*, *MKI67*, and *BTN3A2*), normalized by three reference genes (*CTBP1*, *CUL1*, and *UBQLN1*), and two clinical variables (tumor size and pN status). Patients were categorized as high risk for recurrence or distant metastasis if the BCT score was ≥4, whereas patients with a BCT score <4 were categorized as low risk.

### Propensity Score Matching (PSM)

We used PSM with a 1:1 ratio to match control and treatment groups and enable us to evaluate causal treatment effects by excluding the effects of confounding factors (24). This analysis was performed using a nearest-neighbor matching algorithm in the “MatchIt” package for R software. The propensity score of the cohort was calculated using clinicopathological factors that significantly affect survival (*P* < 0.05 in Cox proportional hazard analysis), and the caliper was set to 0.04.

### Statistical Analysis

Patient characteristics between the groups were compared using independent t-tests for continuous variables and the chi-square or Fisher’s exact test for categorical variables. Disease-free survival (DFS) was defined as the time from the date of surgery to the date of any recurrence, including locoregional recurrence and distant metastasis of breast cancer, and distant metastasis-free survival (DMFS) was defined as the time between the date of surgery and the date of distant metastasis. The probability of DFS and DMFS were estimated using the Kaplan-Meier method, and the log-rank test was used to assess statistical differences in survival rates between groups. Univariate and multivariate analyses were performed using Cox regression and proportional hazard models to evaluate the association between the clinical variables or BCT score and patient outcomes. All hazard ratios are reported with 95% confidence intervals (CIs). All statistical tests were two-sided, and a *P* < 0.05 was regarded as statistically significant. All statistical analyses were performed using SAS 9.4 (SAS Institute, Cary, NC, USA) and R 3.6.2 (http://www.R-project.org).

## Results

### Patient Characteristics

**Figure 1:**
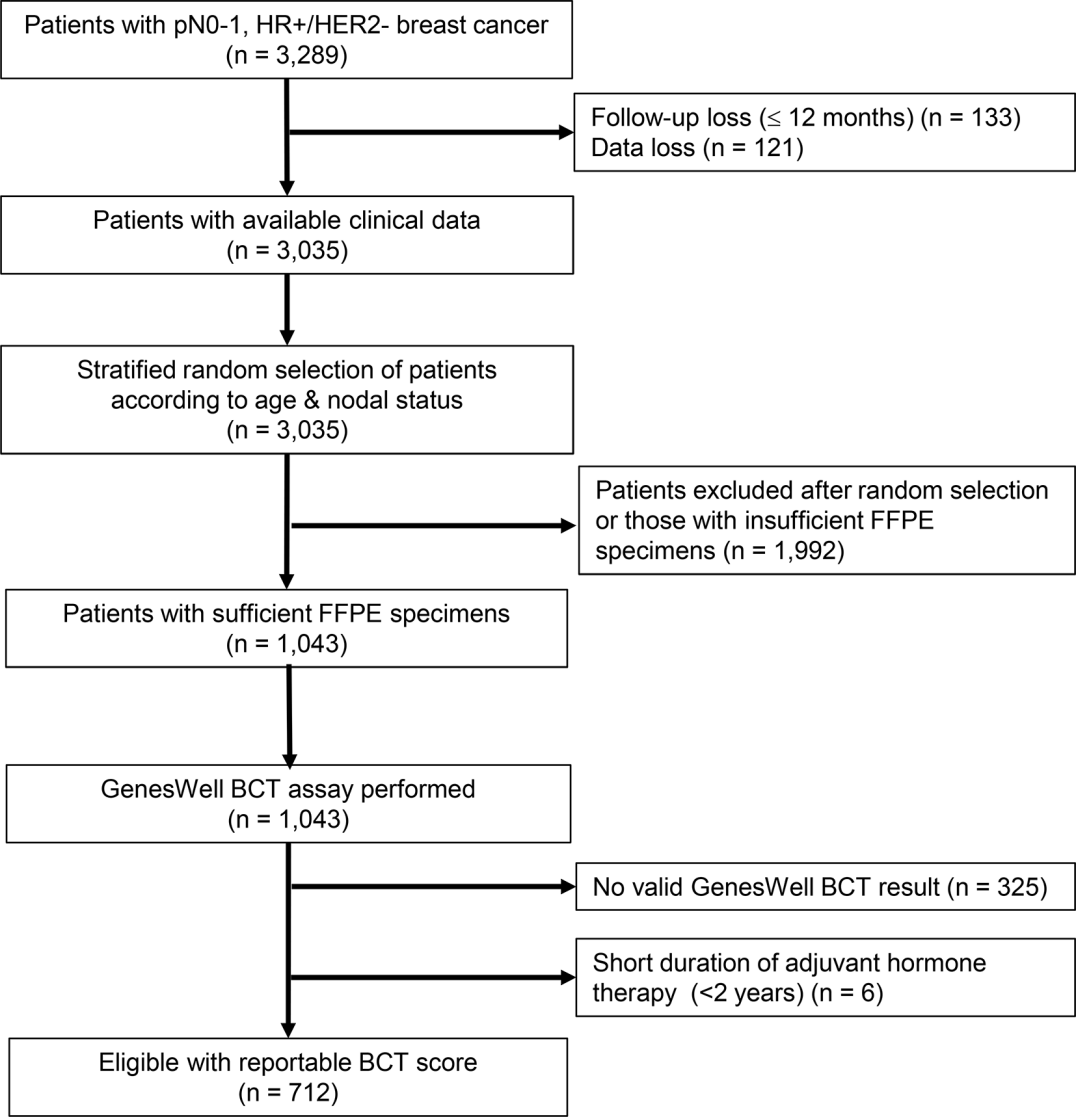
Study flow diagram. SMC, Samsung Medical Center; KNUH, Kyungpook National University Hospital; HR, hormone receptor; HER2, human epidermal growth factor receptor 2; FFPE, formalin-fixed, paraffin-embedded.

Of the 1,043 FFPE samples on which the GenesWell BCT assay was performed, 325 samples that returned invalid GenesWell BCT results and six samples from patients receiving hormone therapy <2 years were excluded. We thus used 712 patients with valid BCT scores in our analyses (**Figure 1**).

The patient characteristics are presented in **Table 1**. The median age of the 712 patients was 48.5 years (range 21–80), and 56.7% of patients (n = 404) were aged ≤50 years. Four hundred thirty-eight (61.5%) patients had pN0 tumors, and 434 (61.0%) patients had small tumors ≤2.0 cm. The median follow-up duration was 7.47 years (range 1.12–13.05). Sixty-six percent of patients were treated with hormone therapy plus chemotherapy, whereas 34.0% of patients received hormone therapy alone. When we compared the clinical characteristics of patients by age group, we found no significant differences in tumor size, pN, nuclear grade, histologic grade, or Ki-67 between age groups (**Table 1**). However, young women with breast cancer were more likely to have a higher rate of LVI (*P* = 0.019) and receive chemotherapy (*P* < 0.001) than older patients (**Table 1**). The incidence of distant metastasis and recurrence in all patients was 5.8 and 9.6%, respectively. Younger patients showed significantly lower 10-year DFS rates than older patients (*P* = 0.020). In contrast, 10-year DMFS did not differ significantly between the age groups (*P* = 0.200). The 10-year DFS rate of patients aged ≤30 years was 65.2%, whereas patents aged >30 years had a >80% of 10-year DFS rate.

**Table 1:** Patient characteristics by age group.

Characteristics	All patients	Age group, years					*P* value	Age group, years		*P* value
		≤30	31–40	41–50	51–60	>60		≤50	>50	
n (%)	712 (100%)	54 (7.6%)	174 (24.4%)	176 (24.7%)	140 (19.7%)	168 (23.6%)		404 (56.7%)	308 (43.3%)	
**Tumor size, cm**							0.331			0.461
≤ 2.0	434 (61.0%)	32 (59.3%)	99 (56.9%)	110 (62.5%)	94 (67.1%)	99 (58.9%)		241 (59.7%)	193 (62.7%)	
> 2.0	278 (39.0%)	22 (40.7%)	75 (43.1%)	66 (37.5%)	46 (32.9%)	69 (41.1%)		163 (40.3%)	115 (37.3%)	
**pN**							0.779			0.531
0	438 (61.5%)	30 (55.6%)	105 (60.3%)	109 (61.9%)	85 (60.7%)	109 (64.9%)		244 (60.4%)	194 (63.0%)	
1	274 (38.5%)	24 (44.4%)	69 (39.7%)	67 (38.1%)	55 (39.3%)	59 (35.1%)		160 (39.6%)	114 (37.0%)	
**Multiplicity**							0.055			**0.008**
Yes	160 (22.5%)	14 (25.9%)	50 (28.7%)	42 (23.9%)	27 (19.3%)	27 (16.1%)		106 (26.2%)	54 (17.5%)	
No	552 (77.5%)	40 (74.1%)	124 (71.3%)	134 (76.1%)	113 (80.7%)	141 (83.9%)		298 (73.8%)	254 (82.5%)	
**LVI**							**0.019**			**0.030**
Yes	231 (32.4%)	26 (48.1%)	66 (37.9%)	53 (30.1%)	40 (28.6%)	46 (27.4%)		145 (35.9%)	86 (27.9%)	
No	481 (67.7%)	28 (51.9%)	108 (62.1%)	123 (69.9%)	100 (71.4%)	122 (72.6%)		259 (64.1%)	222 (72.1%)	
**Stage**							0.665			1.000
I	320 (44.9%)	25 (46.3%)	71 (40.8%)	86 (48.9%)	64 (45.7%)	74 (44.0%)		182 (45.0%)	138 (44.8%)	
II & IIIa	392 (55.1%)	29 (53.7%)	103 (59.2%)	90 (51.1%)	76 (54.3%)	94 (56.0%)		222 (55.0%)	170 (55.2%)	
**Nuclear grade**										0.638
1	132 (18.5%)	10 (18.5%)	24 (13.8%)	38 (21.6%)	28 (20.0%)	32 (19.0%)	0.461	72 (17.8%)	60 (19.5%)	
2	428 (60.1%)	30 (55.6%)	104 (59.8%)	107 (60.8%)	83 (59.3%)	104 (61.9%)		241 (59.7%)	187 (60.7%)	
3	152 (21.3%)	14 (25.9%)	46 (26.4%)	31 (17.6%)	29 (20.7%)	32 (19.0%)		91 (22.5%)	61 (19.8%)	
**Histologic grade**							0.179			0.684
1	234 (32.9%)	11 (20.4%)	52 (29.9%)	70 (39.8%)	43 (30.7%)	58 (34.5%)		133 (32.9%)	101 (32.8%)	
2	350 (49.2%)	32 (59.3%)	84 (48.3%)	80 (45.5%)	74 (52.9%)	80 (47.6%)		196 (48.5%)	154 (50.0%)	
3	124 (17.4%)	11 (20.4%)	38 (21.8%)	26 (14.8%)	21 (15.0%)	28 (16.7%)		75 (18.6%)	49 (15.9%)	
**Ki-67, %**							0.105			0.061
≤20.0	398 (55.9%)	20 (37.0%)	87 (50.0%)	101 (57.4%)	82 (58.6%)	106 (63.1%)		210 (52.0%)	188 (61.0%)	
>20.0	287 (40.3%)	22 (40.7%)	79 (45.4%)	74 (42.0%)	58 (41.4%)	56 (33.3%)		173 (42.8%)	114 (37.0%)	
Unknown	27 (3.8%)	12 (22.2%)	8 (4.6%)	1 (0.6%)	0 (0.0%)	6 (3.6%)		21 (5.2%)	6 (1.9%)	
**Chemotherapy**							**<0.001**			**<0.001**
Yes	470 (66.0%)	41 (75.9%)	140 (80.5%)	130 (73.9%)	92 (65.7%)	67 (39.9%)		311 (77.0%)	159 (51.6%)	
No	242 (34.0%)	13 (24.1%)	34 (19.9%)	46 (26.1%)	48 (34.3%)	101 (60.1%)		93 (23.0%)	149 (48.4%)	
**Distant metastasis**							0.225			0.939
Distant metastasis	41 (5.8%)	2 (3.7%)	16 (9.2%)	6 (3.4%)	7 (5.0%)	10 (6.0%)		24 (5.9%)	17 (5.5%)	
No distant metastasis	671 (94.2%)	52 (96.3%)	158 (90.8%)	170 (96.6%)	133 (95.0%)	158 (94.0%)		380 (94.1%)	291 (94.5%)	
**Recurrence**							**0.033**			0.314
Recurrence	68 (9.6%)	7 (13.0%)	26 (14.9%)	10 (5.7%)	10 (7.1%)	15 (8.9%)		43 (10.6%)	25 (8.1%)	
No recurrence	644 (90.4%)	47 (87.0%)	148 (85.1%)	166 (94.3%)	130 (92.9%)	153 (91.1%)		361 (89.4%)	283 (91.9%)	
**10-year DMFS, % (95% CI)**	91.7% (89.0–94.5%)	93.4% (84.7–100.0%)	87.4% (81.2–94.0%)	96.0% (93.0–99.2%)	94.4% (90.4–98.6%)	87.5% (78.7–97.3%)	0.200	91.9% (88.5–95.3%)	91.4% (86.9–96.2%)	1.000
**10-year DFS, %** **(95% CI)**	86.3% (82.6–90.1%)	65.2% (40.6–100.0%)	80.2% (72.3–89.0%)	93.7% (89.9–97.6%)	92.2% (87.6–97.0%)	82.6% (72.9–93.6%)	**0.020**	85.4% (80.7–90.4%)	88.0% (82.8–93.4%)	0.500

CI, confidence interval; DFS, disease-free survival; DMFS, distant metastasis-free survival; LVI, lymphovascular invasion; pN, pathologic nodal status

P values < 0.05 are marked in bold.

### Prognostic Validation of the BCT Score

**Figure 2:**
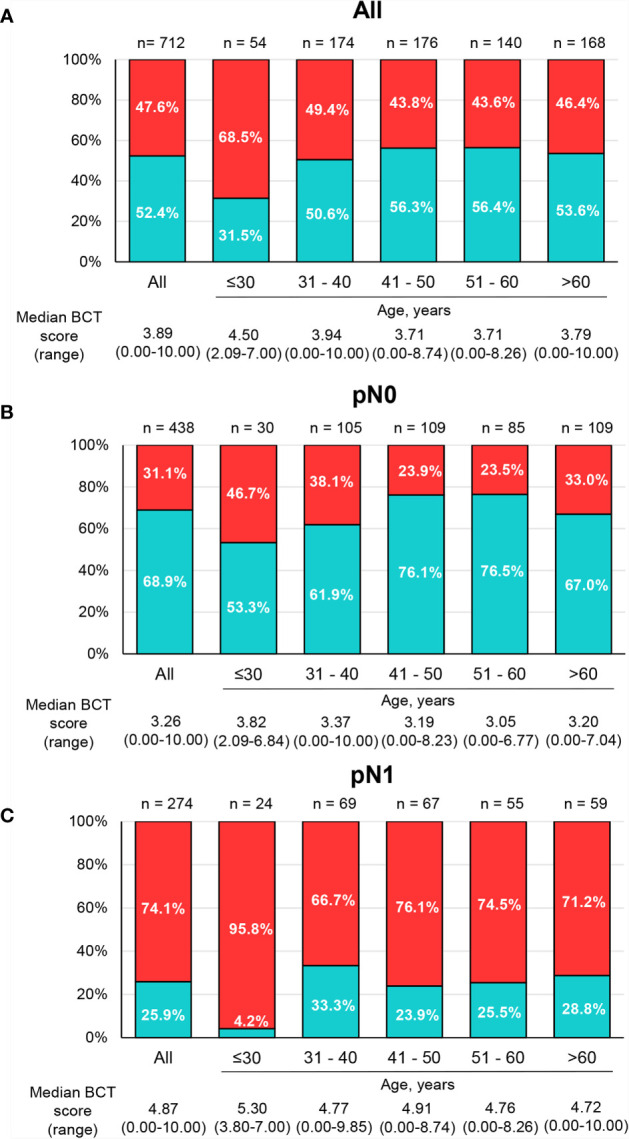
Distribution of BCT scores and risk groups by age group. The percentage of patients within each BCT risk group (blue for the BCT low-risk group and red for the BCT high-risk group) among **(A)** all patients (n = 712), **(B)** pN0 patients (n = 438), and **(C)** pN1 patients (n = 274) by age group. The median BCT score of each age group is also depicted.

The median BCT score of all patients was 3.89 (range 0–10.00), with 52.4% of patients categorized into the BCT low-risk group and 47.6% of patients classified as high risk (**Figure 2A**). The BCT high-risk group was significantly associated with unfavorable clinicopathological factors, including larger tumor size and advanced pN status (**Supplementary Table 1**).

**Figure 3:**
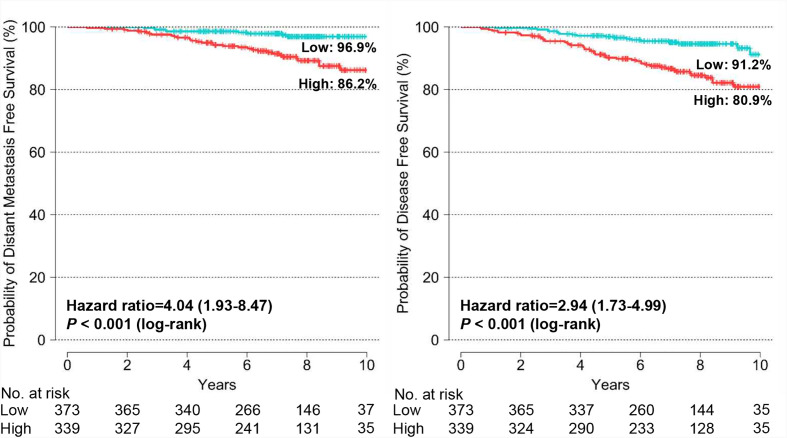
Kaplan-Meier estimates of 10-year distant metastasis-free survival and disease-free survival by BCT risk group in all patients. Patients were classified into the low-risk (blue) or high-risk (red) group according to their BCT scores.

The Kaplan-Meier survival curves show statistically significant differences in DMFS (*P* < 0.001) and DFS (*P* < 0.001) between the BCT low-risk and high-risk groups (**Figure 3**). The probability of 10-year DMFS for patients in the low-risk and high-risk groups was 96.9 and 86.2%, respectively. Recurrence rates at 10 years in patients categorized by the BCT as low risk and high risk were 8.8 and 19.1%, respectively.

We examined the association between the BCT score and patient survival using Cox’s proportional hazard model. In the univariate analysis for DMFS and DFS, the BCT high-risk group was significantly associated with an increased risk of distant metastasis and recurrence (*P* < 0.001) (**Table 2**). A high BCT score also correlated with an increased risk of recurrence (*P* < 0.001). The prognostic significance of the BCT risk group and BCT score was retained in the multivariate analysis. Being in the BCT high-risk group was an independent negative prognostic factor for DMFS (hazard ratio, 2.31; 95% CI, 1.04–5.13; *P* = 0.039) and DFS (hazard ratio, 2.25; 95% CI, 1.26–4.00; *P* = 0.006) (**Table 2**). The BCT score taken as a continuous variable was also independently associated with the risk of recurrence.

**Table 2:** Univariate and multivariate analyses for DMFS and DFS in all patients.

Univariate analysis	DMFS			DFS		
	Hazard ratio	95% CI	*P* value	Hazard ratio	95% CI	*P* value
BCT risk group (Low *vs.* High)	4.04	1.93–8.47	**<0.001**	2.94	1.73–4.99	**<0.001**
BCT score (0–10)	1.38	1.19–1.61	**<0.001**	1.33	1.18–1.50	**<0.001**
Positive node (0–3)	1.75	1.33–2.30	**<0.001**	1.29	1.02–1.63	**0.034**
Tumor size (cm)	1.13	1.03–1.25	**0.013**	1.09	0.99–1.20	0.093
Histologic grade (1 *vs.* 2/3)	3.62	1.42–9.24	**0.007**	3.34	1.66–6.75	**0.001**
**Multivariate analysis**	**DMFS**			**DFS**		
**(BCT risk group)**	**Hazard ratio**	**95% CI**	***P* value**	**Hazard ratio**	**95% CI**	***P* value**
BCT risk group (Low *vs.* High)	2.31	1.04–5.13	**0.039**	2.25	1.26–4.00	**0.006**
Positive node (0–3)	1.46	1.07–2.00	**0.017**	1.06	0.81–1.38	0.674
Tumor size (cm)	1.08	0.92–1.26	0.346	1.04	0.89–1.21	0.575
Histologic grade (1 *vs.* 2/3)	2.76	1.06–7.20	**0.038**	2.69	1.32–5.49	**0.007**
**Multivariate analysis**	**DMFS**			**DFS**		
**(BCT score)**	**Hazard ratio**	**95% CI**	***P* value**	**Hazard ratio**	**95% CI**	***P* value**
BCT score (0–10)	1.24	1.00–1.54	0.051	1.35	1.14–1.60	**0.001**
Positive node (0–3)	1.45	1.06–1.99	**0.021**	1.01	0.77–1.32	0.952
Tumor size (cm)	1.01	0.83–1.23	0.883	0.94	0.78–1.13	0.491
Histologic grade (1 *vs.* 2/3)	2.77	1.07–7.13	**0.035**	2.70	1.33–5.48	**0.006**

CI, confidence interval; DFS, disease-free survival; DMFS, distant metastasis-free survival.

P values < 0.05 are marked in bold.

We also assessed the prognostic value of the BCT score in subgroups of patients divided by treatment and pN status. The BCT score was prognostic for DMFS and DFS among patients treated with hormone therapy plus chemotherapy (**Supplementary Figure 1**). The BCT high-risk group had significantly shorter DMFS (*P* = 0.005) and DFS (*P* = 0.005) than the BCT low-risk group. The BCT score was also prognostic for DFS in patients treated with hormone therapy alone (*P* = 0.002), but it was not prognostic for DMFS in that group. The subgroup analysis by pN status revealed that the BCT score was more prognostic in patients with pN0 tumors than in those with pN1 tumors (**Supplementary Figure 2**). There was a significant difference in DMFS (*P* = 0.040) and DFS (*P* = 0.004) between the BCT low-risk and high-risk groups in patients with pN0 tumors. For patients with pN1 tumors, the BCT score was prognostic for DFS (*P* = 0.030) but not DMFS.

### Prognostic Value of the BCT Score by Age Group

The distribution of BCT scores and risk classification by age group are shown in **Figure 2**. The percentage of young patients categorized into the BCT high-risk group was significantly higher than the percentage of older patients (*P* = 0.019). The median BCT score in younger patients was also significantly higher than that in older patients (*P* = 0.009). In particular, very young patients (aged ≤30 years) had a higher median BCT score (4.50) and a higher likelihood of being in the BCT high-risk group (68.5%) than those in other age groups (**Figure 2A**). Similar results were observed in patients with pN0 tumors and those with pN1 tumors (**Figures 2B, C**). However, the BCT risk classifications and median BCT score did not differ significantly between patients aged ≤50 years and >50 years (**Supplementary Figure 3**).

**Figure 4:**
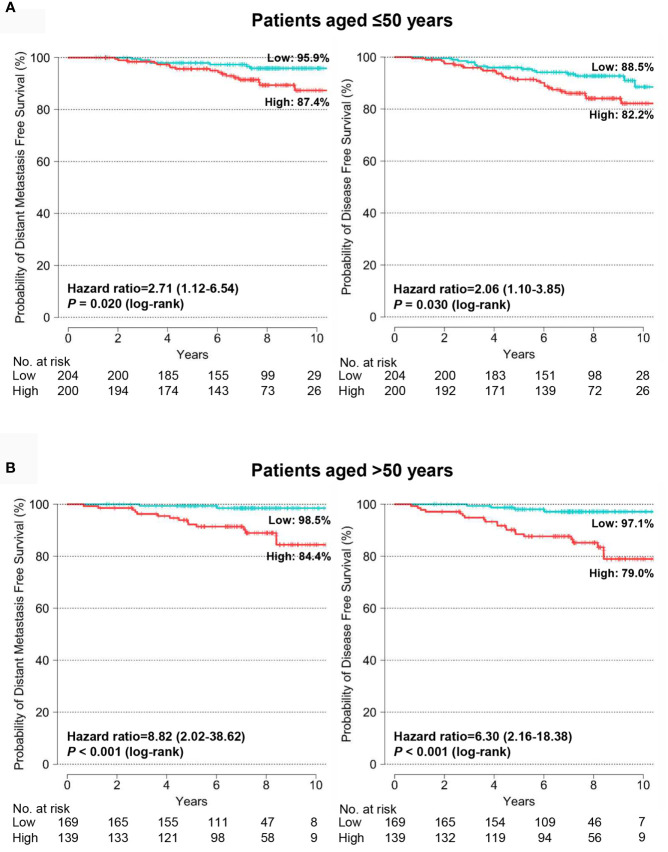
Kaplan-Meier estimates of 10-year distant metastasis-free survival and disease-free survival by BCT risk group in **(A)** patients aged ≤50 years (n = 404) and **(B)** patients aged >50 years (n = 308). Patients were classified into the low-risk (blue) or high-risk (red) group according to their BCT scores.

We assessed the prognostic value of the BCT score in patients aged ≤50 years and >50 years and found that the BCT score was prognostic in both age groups. Kaplan-Meier survival curves showed that the BCT high-risk group had a significantly shorter DMFS and DFS than the BCT low-risk group in both age groups (*P* < 0.001 for DMFS and *P* < 0.001 for DFS in patients aged >50 years; *P* = 0.020 for DMFS and *P* = 0.030 for DFS in patients aged ≤50 years) (**Figure 4**). A high BCT score was significantly associated with an increased risk of recurrence or distant metastasis in patients aged ≤50 years and >50 years (**Table 3**). Moreover, in both age groups, a high BCT score was an independent negative prognostic factor for recurrence (hazard ratio, 1.64; 95% CI, 1.10–2.46; *P* = 0.016 in patients aged >50 years and hazard ratio, 1.28; 95% CI, 1.05–1.56; *P* = 0.015 in patients aged ≤50 years) (**Table 3**).

**Table 3:** Univariate and multivariate analyses for DMFS and DFS in patients aged ≤50 years and >50 years.

Univariate analysis	Patients aged ≤50 years (n = 404)						Patients aged >50 years (n = 308)					
	DMFS			DFS			DMFS			DFS		
	Hazard ratio	95% CI	*P* value	Hazard ratio	95% CI	*P* value	Hazard ratio	95% CI	*P* value	Hazard ratio	95% CI	*P* value
BCT risk group (Low *vs.* High)	2.71	1.12–6.54	**0.026**	2.06	1.10-3.85	**0.024**	8.82	2.02-38.62	**0.004**	6.30	2.16-18.38	**0.001**
BCT score (0–10)	1.33	1.10-1.62	**0.004**	1.25	1.07-1.46	**0.004**	1.48	1.17-1.87	**0.001**	1.48	1.23-1.80	**<0.001**
Positive node (0–3)	1.61	1.13-2.29	**0.009**	1.13	0.83-1.53	0.434	1.98	1.28-3.05	**0.002**	1.58	1.09-2.30	**0.016**
Tumor size (cm)	1.11	0.98-1.25	0.101	1.03	0.89-1.19	0.695	1.26	1.03-1.55	**0.029**	1.26	1.06-1.49	**0.007**
Histologic grade (1 *vs.* 2/3)	3.76	1.12–12.61	**0.032**	3.29	1.39-7.80	**0.007**	3.50	0.79-15.39	0.098	3.55	1.06-11.90	**0.040**
**Multivariate analysis**	**DMFS**			**DFS**			**DMFS**			**DFS**		
**(BCT risk group)**	**Hazard ratio**	**95% CI**	***P* value**	**Hazard ratio**	**95% CI**	***P* value**	**Hazard ratio**	**95% CI**	***P* value**	**Hazard ratio**	**95% CI**	***P* value**
BCT risk group (Low *vs.* High)	1.66	0.64–4.32	0.297	1.74	0.88–3.47	0.113	4.79	0.98–23.42	0.053	4.04	1.26–12.96	**0.019**
Positive node (0–3)	1.36	0.90–2.05	0.140	0.98	0.69–1.38	0.895	1.62	0.99–2.66	0.055	1.21	0.79–1.84	0.374
Tumor size (cm)	1.08	0.90–1.29	0.398	0.99	0.81–1.22	0.956	1.03	0.75–1.42	0.851	1.09	0.87–1.38	0.449
Histologic grade (1 *vs.* 2/3)	3.22	0.93–11.16	0.066	2.89	1.20–6.94	**0.018**	2.19	0.49–9.83	0.307	2.36	0.69–8.07	0.170
**Multivariate analysis**	**DMFS**			**DFS**			**DMFS**			**DFS**		
**(BCT score)**	**Hazard ratio**	**95% CI**	***P* value**	**Hazard ratio**	**95% CI**	***P* value**	**Hazard ratio**	**95% CI**	***P* value**	**Hazard ratio**	**95% CI**	***P* value**
BCT score (0–10)	1.21	0.94–1.54	0.139	1.28	1.05–1.56	**0.015**	1.44	0.87–2.39	0.151	1.64	1.10–2.46	**0.016**
Positive node (0–3)	1.31	0.87–1.99	0.194	0.93	0.65–1.31	0.666	1.61	0.96–2.72	0.072	1.12	0.72–1.76	0.615
Tumor size (cm)	1.04	0.84–1.30	0.692	0.92	0.74–1.16	0.501	0.88	0.55–1.40	0.578	0.85	0.59–1.23	0.388
Histologic grade (1 *vs.* 2/3)	3.08	0.91–10.45	0.071	2.89	1.21–6.89	**0.017**	2.18	0.48–9.95	0.315	2.32	0.68–7.89	0.178

CI, confidence interval; DFS, disease-free survival; DMFS, distant metastasis-free survival.

P values < 0.05 are marked in bold.

### Predictive Value of the BCT Score for Chemotherapy Benefit in the PSM Cohort

**Figure 5:**
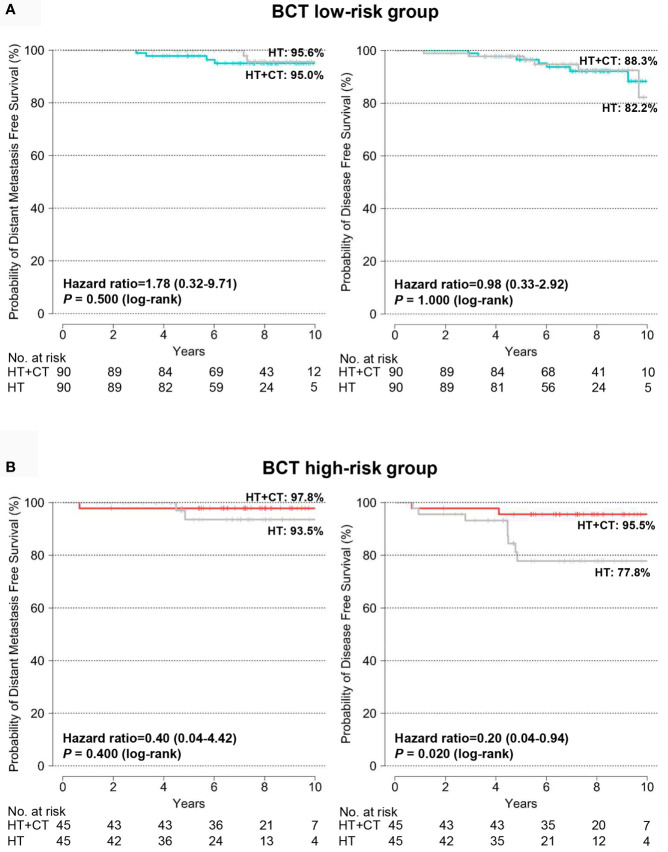
Kaplan-Meier estimates of 10-year distant metastasis-free survival and disease-free survival according to treatment group in the PSM cohort. **(A)** BCT low-risk (n = 180) and **(B)** BCT high-risk (n = 90) group. Patients were treated with either hormone therapy alone (HT) or hormone therapy plus chemotherapy (HT+CT). PSM, Propensity score matching.

In the original cohort, clinicopathological characteristics differed significantly between treatment groups (hormone therapy alone vs. hormone therapy plus chemotherapy) within the BCT risk groups. Patients treated with hormone therapy plus chemotherapy had unfavorable clinicopathological status, including larger tumor size, higher histologic grade, and advanced pN status, compared with those treated with hormone therapy alone in both the BCT low-risk and high-risk groups (**Supplementary Table 2**). Therefore, it was not feasible to assess the predictive value of the BCT score for chemotherapy benefit in the original cohort (**Supplementary Figure 4**). Using the PSM method, we generated a matched cohort in which the clinical characteristics did not differ significantly between treatment groups (**Supplementary Table 2**). In the PSM cohort, the 10-year DFS for the BCT high-risk group (n = 90) improved significantly, from 77.8 to 95.5%, after the addition of chemotherapy to hormone therapy (hazard ratio, 0.20; 95% CI, 0.04–0.94; *P* = 0.020) (**Figure 5**). In contrast, 10-year DFS did not differ significantly between the two treatment groups in the BCT low-risk group (n = 180) (**Figure 5**).

## Discussion

Following a previous study (20), this study further validated the prognostic ability of the BCT score to predict recurrences in patients with HR+/HER2− early breast cancer according to age group in an independent cohort. DMFS and DFS differed significantly between the BCT low-risk and high-risk groups, and being in the BCT high-risk group was an independent negative prognostic factor for both DMFS and DFS. Importantly, our subgroup analysis by age group demonstrated that the BCT score was prognostic in patients aged ≤50 years, whereas it had more prognostic value in patients aged >50 years. These results suggest that the BCT score is prognostic irrespective of age. However, although using the BCT score as a continuous variable or a risk group indicator retained its significance for DFS in the multivariate analysis, the BCT score was not independently associated with DMFS in patients aged ≤50 years or >50 years. This finding might be attributable to the very low rate of distant metastasis in this study (5.8%) compared with that found in a previous study (13.1%) (20) due to our short follow-up period or to the treatment effect of chemotherapy, particularly in the BCT high-risk group. Most patients (83.8%, 284/339) classified into the BCT high-risk group received adjuvant chemotherapy, which could have affected the patient outcomes. To validate the prognostic value of the BCT score for DMFS in each age group, a longer follow-up to this study or an additional study with more patients will be needed.

Multigene assays, such as MammaPrint and Oncotype DX, are commonly used to predict the prognosis of early breast cancer patients of all ages, but their prognostic or predictive value in young patients is unclear. Moreover, some assays, such as Prosigna and EndoPredict, are indicated or validated only for use in postmenopausal patients (11, 25–28); only a few studies have tested their prognostic significance in premenopausal patients. EndoPredict was shown to be prognostic in premenopausal patients who are node-positive and have received chemotherapy (29). A recent study also showed that continuous Prosigna ROR scores were prognostic in high-risk premenopausal patients, most of whom were lymph node–positive and received cyclophosphamide-based adjuvant chemotherapy (30). Given that young patients are more likely to receive chemotherapy than older patients, as confirmed in this study, it is notable that the BCT score can be used to avoid unnecessary chemotherapy in young patients who are likely to receive aggressive therapy by accurately identifying low-risk patients who will not benefit from chemotherapy.

When we compared the BCT score distribution and clinicopathological parameters according to age group, we found that younger patients had tumors with higher LVI and that a higher percentage of young patients was classified into the BCT high-risk group than older patients. In particular, very young patients (aged ≤30 years) composed the highest percentage of the BCT high-risk group and had shorter DFS than patients of other ages, indicating that very young patients with HR+/HER2− early breast cancer have a poorer prognosis than older patients. These results are in line with previous studies, which showed that the prognosis of patients aged <35 years with HR+ breast cancer is worse than that of patients aged 35–50 years (3) and that patients in their 20s who had HR+ breast cancer had significantly worse outcomes than those in 30s and 40s (2). A larger difference between DMFS and DFS in patients aged ≤30 years compared with older age groups was observed in this study. This difference might be due to a shorter follow-up period of the youngest subgroup than that of other age groups (median follow-up duration, 4.95 years *vs.* 6.82 to 7.97 years). Similar to a previous study (21), we found that the distribution of BCT scores between patients aged ≤50 years and those >50 years was similar, whereas patients with pN1 tumors formed a higher percentage of the BCT high-risk group than patients with pN0 tumors.

In the cohort matched using the PSM method, patients classified as high risk by their BCT scores showed a significant improvement in survival after adding chemotherapy to hormone therapy. In contrast, those at low risk according to their BCT scores received no significant survival benefit from adding chemotherapy. In line with a previous study (31), our results also suggest that the BCT score can predict whether patients with HR+/HER2− early breast cancer will benefit from adding chemotherapy to hormone therapy.

Despite its strengths, this study has some limitations. Many (~30%) FFPE tissue samples could not be evaluated by the GenesWell BCT assay because of significant degradation in the mRNA extracted from FFPE samples stored for longer than 10 years. RNA degradation increases with the storage time of FFPE tissues (32). Moreover, due to a very low rate of distant metastasis in patients included in this study, the BCT score was an independent prognostic factor for DFS, but not for DMFS. This study is a retrospective study; a prospective study to assess the prognostic and predictive of the BCT score is required. For this reason, a randomized prospective trial is being conducted to evaluate 10-year DMFS according to adjuvant chemotherapy in patients classified as clinical high and BCT low risk (ClinicalTrials.gov number NCT04278469).

## Conclusions

This study demonstrated that the BCT score is prognostic in patients aged ≤50 years and those aged >50 years. The BCT score can be used to identify low-risk patients who will not benefit from adjuvant chemotherapy to treat early breast cancer, irrespective of age. A further prospective study to assess the prognostic and predictive value of the BCT score is required.

## Data Availability Statement

The original contributions presented in the study are included in the article/**Supplementary Material**. Further inquiries can be directed to the corresponding author.

## Ethics Statement

This study was approved by the Institutional Review Board (IRB) of Samsung Medical Center (SMC) (Seoul) and Kyungpook National University Hospital (KNUH) (Daegu) (IRB No.: 2017-01-054 and 2017-01-029) in the Republic of Korea and performed in accordance with the Declaration of Helsinki. Because the study is retrospective and patient information was anonymized and de-identified prior to analysis, the requirement for informed consent was waived. A general consent to use the samples for research purpose was obtained from patients at the time of surgery.

## Author Contributions

JEL and YKS conceived the study and participated in its design. JMR, SYC, JL, SJL and J-YP were involved in data acquisition. MJK and JMR drafted the manuscript. MJK, JMR, JH, KK and JEL analyzed and interpreted the data. JH and SH performed statistical analyses. SJN, SWK, and YM provided administrative, technical, or material support. KK supervised the statistical analysis. YKS participated in critical revisions of the manuscript with respect to important intellectual content. HYP and JEL supervised the study. All authors contributed to the article and approved the submitted version.

## Funding

This research was supported by a grant from the Korea Health Technology R&D Project through the Korea Health Industry Development Institute (KHIDI), funded by the Ministry of Health & Welfare, Republic of Korea (HI17C1142) (JEL) and by the National Research Foundation of Korea (NRF) grant, funded by the Korea government (MSIT) (NRF-2020R1A5A2017323) (MJK). The funders had no role in study design, collection, analysis and interpretation of data, preparation of the manuscript or decision to publish.

## Conflict of Interest

JH and YM are employees of Gencurix. Gencurix provided a support in the form of salaries for the authors JH and YM, but did not have any additional role in the study design, data collection and analysis, decision to publish, or preparation of the manuscript.

The remaining authors declare that the research was conducted in the absence of any commercial or financial relationships that could be construed as a potential conflict of interest.
