# Supplementary material for: Validation of the GenesWell BCT Score in Young Asian Women With HR+/HER2− Early Breast Cancer
Source: Front Oncol. 2021 Feb 23;11:588728. doi: 10.3389/fonc.2021.588728 (PMC7942224; doi:10.3389/fonc.2021.588728)
Supplement: Supplementary file 1 [file DataSheet_1.docx]

**Supplementary Material**

**Validation of the GenesWell BCT Score in Young Asian Women with HR+/HER2- Early Breast Cancer**

Mi Jeong Kwon^+^, Jai Min Ryu^+^, Soo Youn Cho, Seok Jin Nam, Seok Won Kim, Jeeyeon Lee, Soo Jung Lee, Ji-Young Park, Ho Yong Park, Sungjun Hong, Kyunga Kim, Jinil Han, Youngho Moon, Young Kee Shin, and Jeong Eon Lee*

^+^ Mi Jeong Kwon and Jai Min Ryu contributed equally to this work.

*** Correspondence:**Jeong Eon Lee
paojlus@hanmail.net

**Supplementary Tables**

**Supplementary Table 1.** Patient characteristics of the risk groups according to the BCT score

|  |  |  |  | **BCT risk group** | |  |
| --- | --- | --- | --- | --- | --- | --- |
| **Characteristics** |  | **All patients** |  | **Low-risk** | **High-risk** | ***P* value** |
| n (%) |  | 712 (100%) |  | 373 (52.4%) | 339 (47.6%) |  |
| **Age group, years** |  |  |  |  |  | 0.279 |
| ≤50 |  | 404 (56.7%) |  | 204 (50.5%) | 200 (49.5%) |  |
| >50 |  | 308 (43.3%) |  | 169 (54.9%) | 139 (45.1%) |  |
| **Tumor size, cm** |  |  |  |  |  | **<0.001** |
| ≤ 2 |  | 434 (61.0%) |  | 304 (70.0%) | 130 (30.0%) |  |
| > 2 |  | 278 (39.0%) |  | 69 (24.8%) | 209 (75.2%) |  |
| **pN** |  |  |  |  |  | **<0.001** |
| 0 |  | 438 (61.5%) |  | 302 (68.9%) | 136 (31.1%) |  |
| 1 |  | 274 (38.5%) |  | 71 (25.9%) | 203 (74.1%) |  |
| **Multiplicity** |  |  |  |  |  | **0.010** |
| Yes |  | 160 (22.5%) |  | 69 (43.1%) | 91 (56.9%) |  |
| No |  | 552 (77.5%) |  | 304 (55.1%) | 248 (44.9%) |  |
| **LVI** |  |  |  |  |  | **<0.001** |
| Yes |  | 231 (32.4%) |  | 84 (36.4%) | 147 (63.6%) |  |
| No |  | 481 (67.7%) |  | 289 (60.1%) | 192 (39.9%) |  |
| **Stage** |  |  |  |  |  | **<0.001** |
| I |  | 320 (44.9%) |  | 261 (81.6%) | 59 (18.4%) |  |
| II & IIIa |  | 392 (55.1%) |  | 112 (28.6%) | 280 (71.4%) |  |
| **Nuclear grade** |  |  |  |  |  | **<0.001** |
| 1 |  | 132 (18.5%) |  | 93 (70.5%) | 39 (29.5%) |  |
| 2 |  | 428 (60.1%) |  | 239 (55.8%) | 189 (44.2%) |  |
| 3 |  | 152 (21.3%) |  | 41 (27.0%) | 111 (73.0%) |  |
| **Histologic grade** |  |  |  |  |  | **<0.001** |
| 1 |  | 234 (32.9%) |  | 164 (70.1%) | 70 (29.9%) |  |
| 2 |  | 350 (49.2%) |  | 177 (50.6%) | 173 (49.4%) |  |
| 3 |  | 124 (17.4%) |  | 31 (25.0%) | 93 (75.0%) |  |
| **Ki67, %** |  |  |  |  |  | **<0.001** |
| ≤20 |  | 398 (55.9%) |  | 260 (65.3%) | 138 (34.7%) |  |
| >20 |  | 287 (40.3%) |  | 98 (34.1%) | 189 (65.9%) |  |
| Unknown |  | 27 (3.8%) |  | 15 (55.6%) | 12 (44.4%) |  |
| **Chemotherapy** |  |  |  |  |  | **<0.001** |
| Yes |  | 470 (66.0%) |  | 186 (39.6%) | 284 (60.4%) |  |
| No |  | 242 (34.0%) |  | 187 (77.3%) | 55 (22.7%) |  |
| **BCT Score, median** |  | 3.89 |  | 2.9 | 5.06 |  |
| **10-year DMFS, % (95% CI)** |  | 91.7% (89.0-94.5%) |  | 96.9% (94.9-9.0%) | 86.2% (81.3-91.4%) | **<0.001** |
| **10-year DFS, %**  **(95% CI)** |  | 86.3% (82.6-90.1%) |  | 91.2% (86.1-96.7%) | 80.9% (75.6-86.6%) | **<0.001** |
| Abbreviations : CI, confidence interval; DFS, disease-free survival; DMFS, distant metastasis-free survival; LVI, lymphovascular invasion; pN, pathologic nodal status  *P* values < 0.05 are marked in bold. | | | | | | |

**Supplementary Table 2.** Patient characteristics in the original cohort and PSM cohort

|  |  | **BCT low-risk group** | | | | | | |  |  | **BCT high-risk group** | | | | | | |  |
| --- | --- | --- | --- | --- | --- | --- | --- | --- | --- | --- | --- | --- | --- | --- | --- | --- | --- | --- |
|  |  | **Original cohort** | | |  | **PSM cohort** | | |  |  | **Original cohort** | | |  | **PSM cohort** | | | |
| **Characteristics** |  | **HT+CT** | **HT alone** | ***P* value** |  | **HT+CT** | **HT alone** | ***P* value** |  |  | **HT+CT** | **HT alone** | ***P* value** |  | **HT+CT** | **HT alone** | ***P* value** | |
| n (%) |  | 186 | 187 |  |  | 90 | 90 |  |  |  | 284 | 55 |  |  | 45 | 45 |  | |
| **Age group, years** | |  |  | **<0.001** |  |  |  | 1.000 |  |  |  |  | **<0.001** |  |  |  | 0.509 | |
| ≤50 |  | 130 (69.9%) | 74 (39.6%) |  |  | 52 (57.8%) | 53 (58.9%) |  |  |  | 181 (63.7%) | 19 (34.5%) |  |  | 14 (31.1%) | 18 (40.0%) |  | |
| >50 |  | 56 (30.1%) | 113 (60.4%) |  |  | 38 (42.2%) | 37 (41.1%) |  |  |  | 103 (36.3%) | 36 (65.5%) |  |  | 31 (68.9%) | 27 (60.0%) |  | |
| **Tumor size, cm** |  |  |  | **<0.001** |  |  |  | 0.666 |  |  |  |  | **0.011** |  |  |  | 1.000 | |
| ≤2 |  | 136 (73.1%) | 168 (89.8%) |  |  | 76 (84.4%) | 79 (87.8%) |  |  |  | 100 (35.2%) | 30 (54.5%) |  |  | 20 (44.4%) | 20 (44.4%) |  | |
| >2 |  | 50 (26.9%) | 19 (10.2%) |  |  | 14 (15.6%) | 11 (12.2%) |  |  |  | 184 (64.8%) | 25 (45.5%) |  |  | 25 (55.6%) | 25 (55.6%) |  | |
| **pN** |  |  |  | **<0.001** |  |  |  | 1.000 |  |  |  |  | **<0.001** |  |  |  | 0.292 | |
| 0 |  | 124 (66.7%) | 178 (95.2%) |  |  | 82 (91.1%) | 81 (90.0%) |  |  |  | 100 (35.2%) | 36 (65.5%) |  |  | 20 (44.4%) | 26 (57.8%) |  | |
| 1 |  | 62 (33.3%) | 9 (4.8%) |  |  | 8 (8.9%) | 9 (10.0%) |  |  |  | 184 (64.8%) | 19 (34.5%) |  |  | 25 (55.6%) | 19 (42.2%) |  | |
| **Histologic grade** |  |  |  | **<0.001** |  |  |  | 1.000 |  |  |  |  | **<0.001** |  |  |  | 0.833 | |
| 1 |  | 59 (31.7%) | 105 (56.1%) |  |  | 30 (33.3%) | 30 (33.3%) |  |  |  | 47 (16.5%) | 23 (41.8%) |  |  | 22 (48.9%) | 20 (44.4%) |  | |
| 2/3 |  | 126 (67.7%) | 82 (43.9%) |  |  | 60 (66.7%) | 60 (66.7%) |  |  |  | 234 (82.4%) | 32 (58.2%) |  |  | 23 (51.1%) | 25 (55.6%) |  | |
| NA |  | 1 (0.5%) | 0 (0.0%) |  |  | 0 (0.0%) | 0 (0.0%) |  |  |  | 3 (1.1%) | 0 (0.0%) |  |  | 0 (0.0%) | 0 (0.0%) |  | |
| **LVI** |  |  |  | **<0.001** |  |  |  | 0.845 |  |  |  |  | 0.059 |  |  |  | 0.086 | |
| Yes |  | 59 (31.7%) | 25 (13.4%) |  |  | 17 (18.9%) | 15 (16.7%) |  |  |  | 130 (45.8%) | 17 (30.9%) |  |  | 7 (15.6%) | 15 (33.3%) |  | |
| No |  | 127 (68.3%) | 162 (86.6%) |  |  | 73 (81.1%) | 75 (83.3%) |  |  |  | 154 (54.2%) | 38 (69.1%) |  |  | 38 (84.4%) | 30 (66.7%) |  | |
| **10-year DMFS, % (95% CI)** |  | 95.9% (93.0-98.9%) | 97.6% (94.3-100.0%) | 0.100 |  | 95.0% (90.3-99.9%) | 95.6% (89.7-100.0%) | 0.500 |  |  | 85.1% (79.7-90.8%) | 94.6% (87.6-100.0%) | 0.200 |  | 97.8% (93.6-100.0%) | 93.5% (85.2-100.0%) | 0.400 | |
| **10-year DFS, % (95% CI)** |  | 91.6% (86.6-96.9%) | 88.3% (75.1-100.0%) | 0.400 |  | 88.3% (79.4-98.2%) | 82.2% (64.6-100.0%) | 1.000 |  |  | 81.0% (75.3-87.2%) | 81.7% (70.8-94.3%) | 0.600 |  | 95.5% (89.6-100.0%) | 77.8% (65.1-93.0%) | **0.020** | |
| Abbreviations : CI, confidence interval; DFS, disease-free survival; DMFS, distant metastasis-free survival; HT, hormone therapy; HT+CT, hormone therapy plus chemotherapy; LVI, lymphovascular invasion; pN, pathologic nodal status; PSM, Propensity score matching  *P* values < 0.05 are marked in bold. | | | | | | | | | | | | | | | | | |  |

**Supplementary Figures**


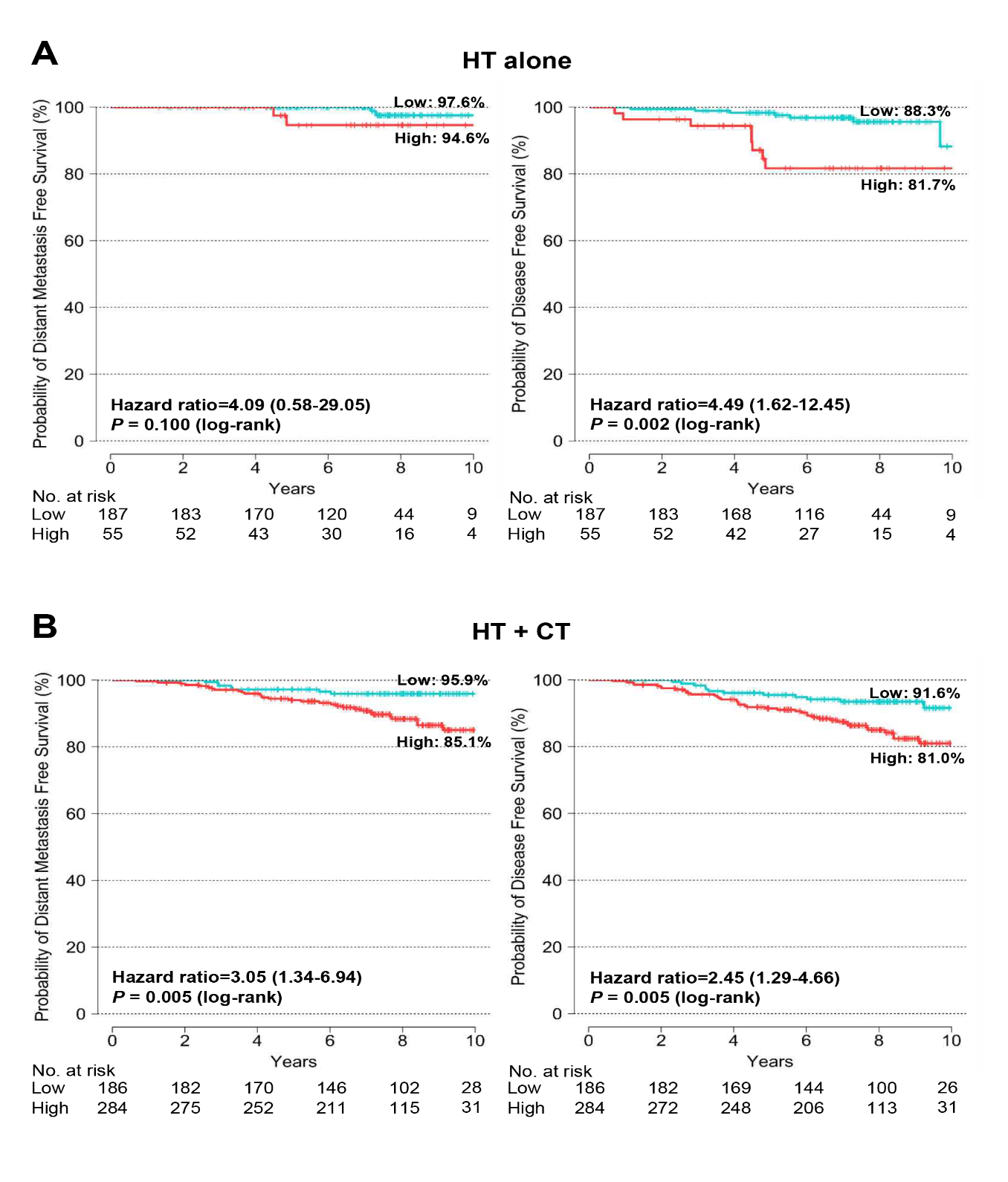


**Supplementary Figure 1.** Kaplan-Meier estimates of 10-year distant metastasis-free survival and disease-free survival by BCT risk group in (**A**) patients treated with hormone therapy (HT) alone (n = 242) and (**B**) those treated with hormone therapy plus chemotherapy (HT+CT) (n = 470).


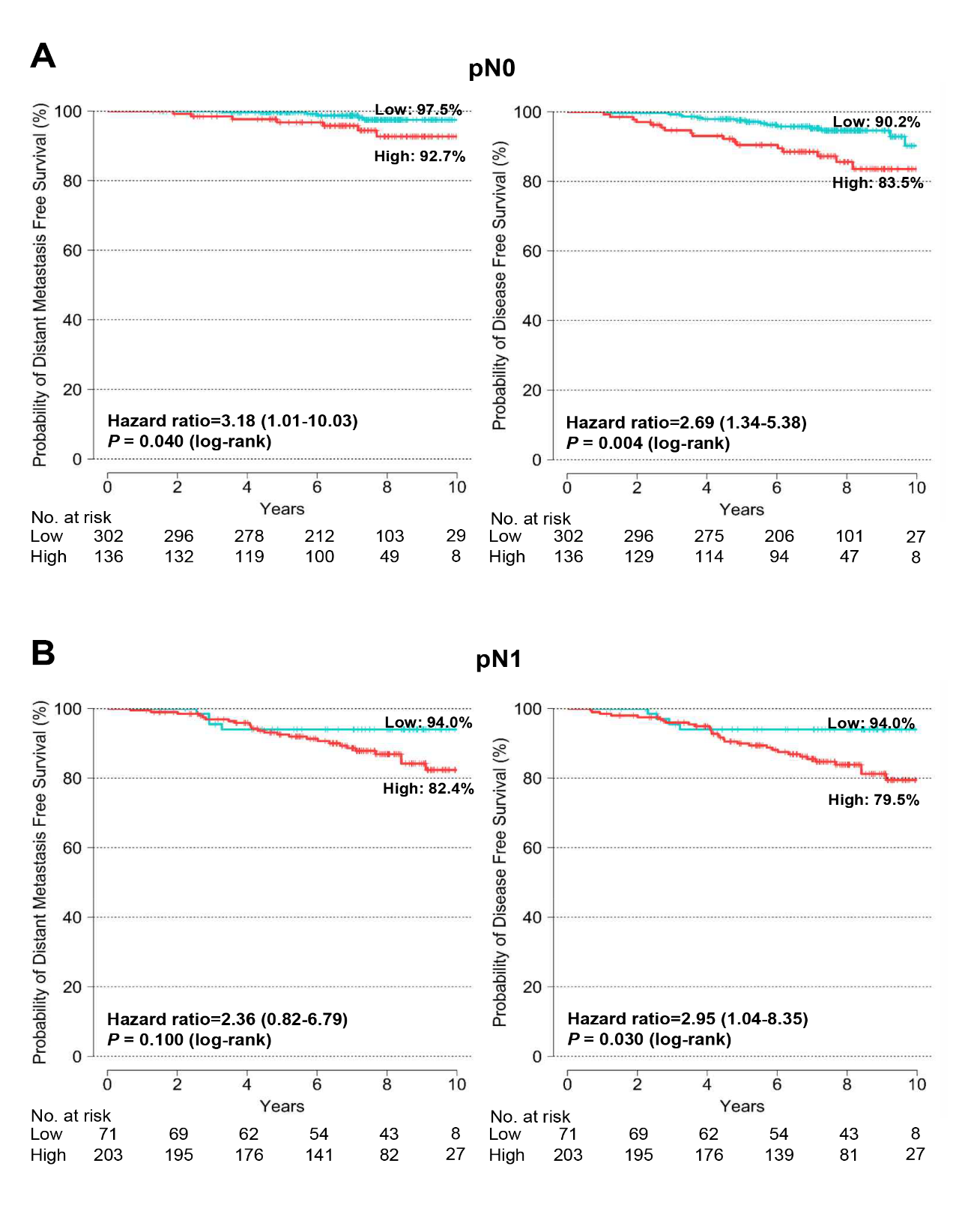


**Supplementary Figure 2.** Kaplan-Meier estimates of 10-year distant metastasis-free survival and disease-free survival by BCT risk group in patients with (**A**) pN0 (n = 438) and (**B**) pN1 tumors (n = 274).

**
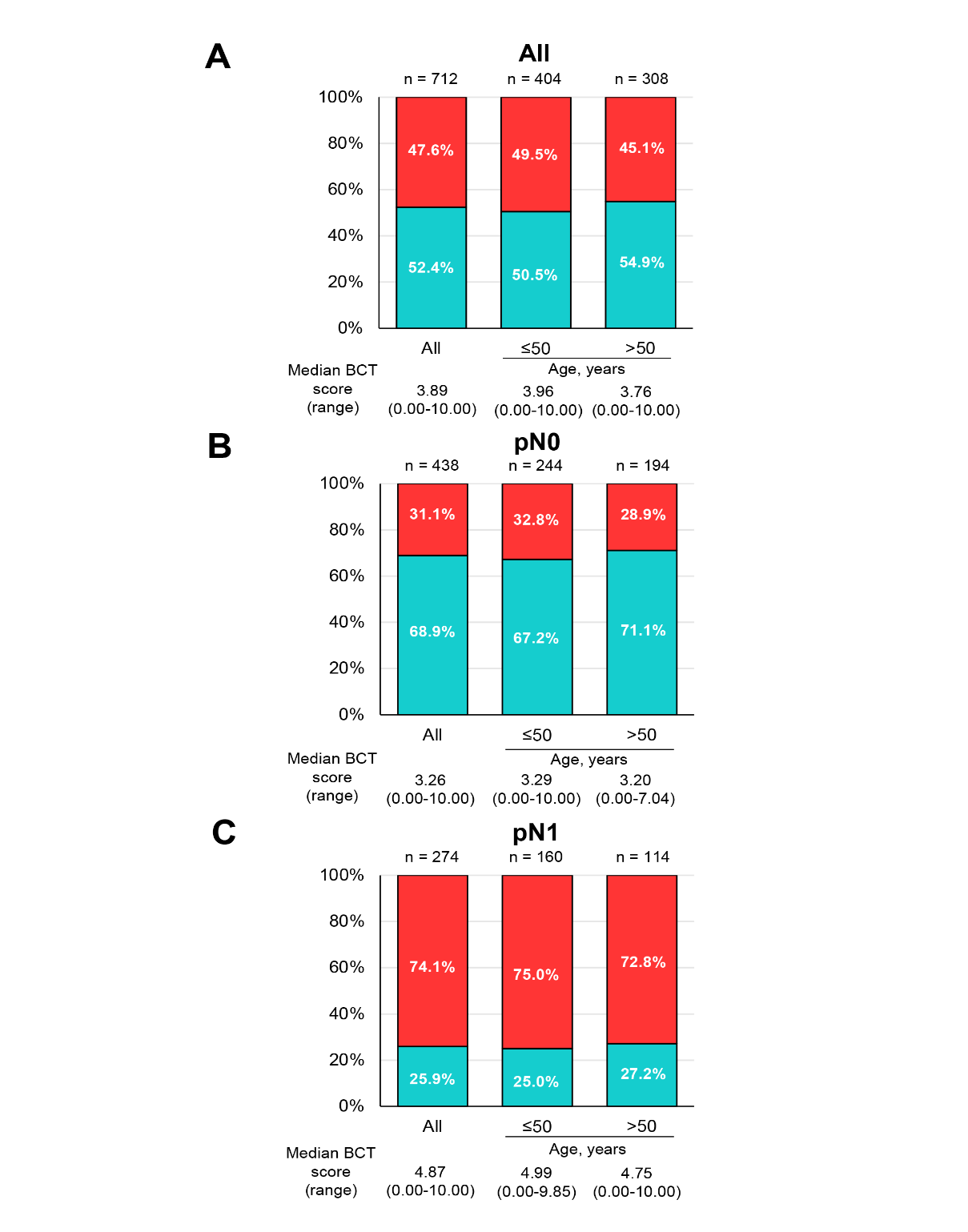
**

**Supplementary Figure 3**. Distribution of the BCT scores and risk groups in patients aged ≤ 50 years and > 50 years. The percentage of patients within each BCT risk group (blue for BCT low-risk group and red for BCT high-risk group) among (**A**) all patients (n = 712), (**B**) patients with pN0 tumors (n = 438) and (**C**) pN1 tumors (n = 274). The median BCT score of each age group is also depicted

**
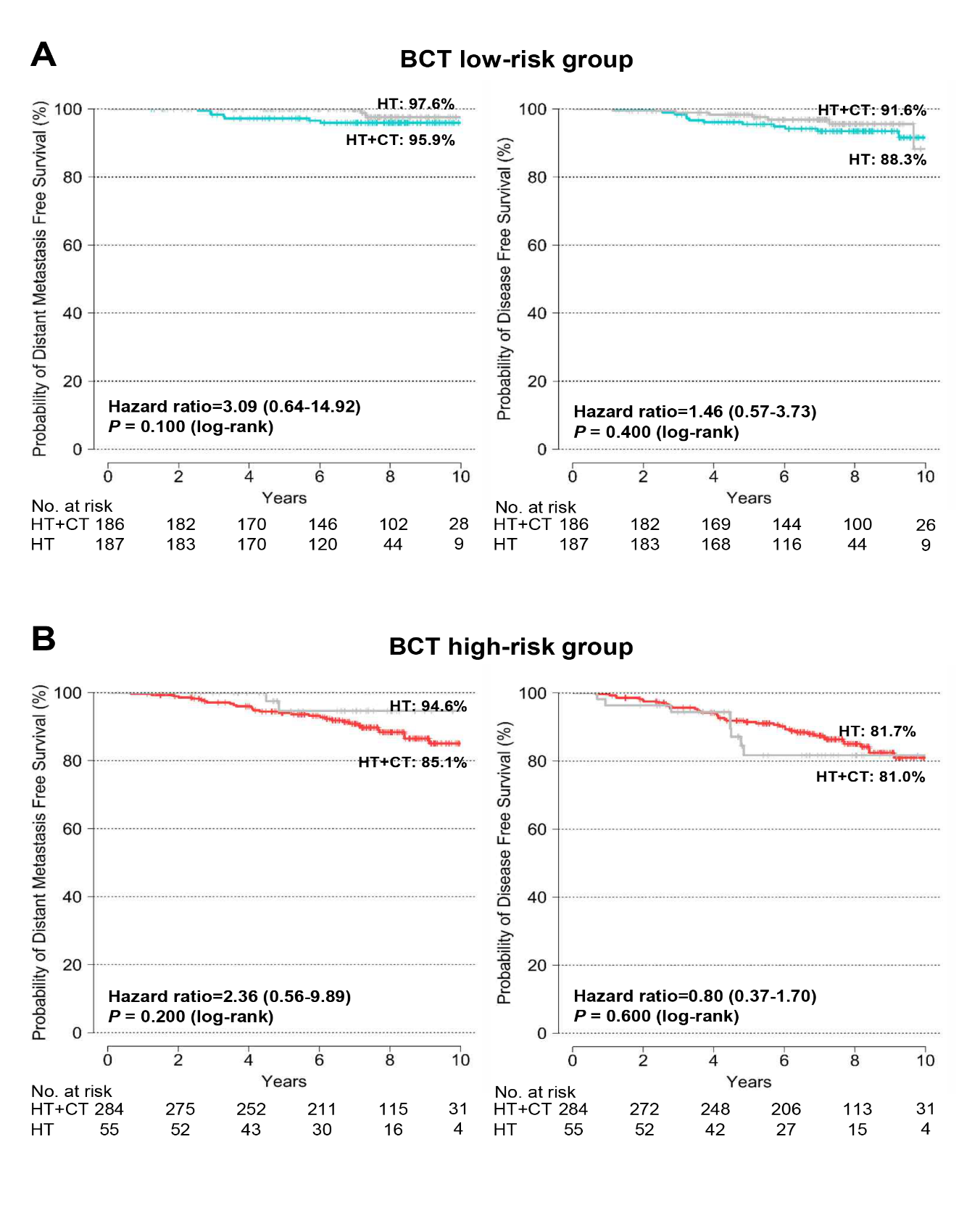
**

**Supplementary Figure 4**. Kaplan-Meier estimates of 10-year distant metastasis-free survival and disease-free survival by treatment group in the original cohort. (**A**) BCT low-risk (n = 373) and (**B**) BCT high-risk (n = 339) group. Patients were treated with either hormone therapy alone (HT) or hormone therapy plus chemotherapy (HT+ CT).
